# 84 A Proof-of-concept for a Continuous-temperature Circulating Water Bath in Frostbite Limb Rewarming

**DOI:** 10.1093/jbcr/iraf019.084

**Published:** 2025-04-01

**Authors:** Robert McKenzie, Joshua Wong, Alexis Armour

**Affiliations:** University of Alberta; University of Alberta; University of Alberta

## Abstract

**Introduction:**

Frostbite treatment is challenging due to tissue damage from ischemia and crystal formation. The American Burn Association advises rapid rewarming in 38-42°C water, yet hospital implementation is inconsistent. Our objective is to facilitate this step of frostbite treatment with a proof-of-concept, descriptive study of a proposed continuous-temperature circulating (CTC) water bath. We hypothesize that this design will effectively rewarm chilled extremities within the requisite 30 minutes without requiring frequent monitoring and adjustment of water temperature.

**Methods:**

We constructed a CTC water bath system with a reservoir and a continuous warming and circulating device (CWCD). Water and tissue temperature were monitored using a needle probe thermometer. Pigs’ feet were chilled and immersed in 39.0 °C water with or without a CWCD.

**Results:**

Without a CWCD, tissue warmed from 3.2 ± 0.3 °C to 34.2 ± 0.2 °C over 30 minutes, with a final water temperature of 36.5 ± 0.1 °C. With a CWCD, tissue warmed from 2.7 ± 0.2 °C to 36.7 ± 0.2 °C, with a final water temperature of 39.1 ± 0.1 °C.

**Conclusions:**

This study demonstrates that the CTC water baths maintained at 39.0 °C offer a reliable and effective method for rewarming hypothermic tissue within 30 minutes. Continuous water temperature at 39.0 °C proved crucial in achieving euthermic tissue warming. These findings suggest that our approach could provide a practical solution to the inconsistent and impractical nature of current frostbite rewarming methods in clinical settings. The highest factor contributing to rewarming compliance is temperature regulation. These are mitigated by the CTC water bath. Further studies are ongoing to validate these findings in clinical practice and explore scalability and feasibility.

**Applicability of Research to Practice:**

This research demonstrates the effective rewarming of chilled tissues using a CTC water bath. Future research will focus on developing a portable CTC water bath for implementation in acute care settings in the initial treatment of frostbite. This device may be utilized further in paramedic vehicles/stations and rural clinics where patients need long transport to the regional burn centers.

**Funding for the Study:**

N/A

